# Supplementary material for: Community health knowledge and access to care in post-conflict Northern Uganda: Perspectives of community health workers in Pader District
Source: PLOS Glob Public Health. 2026 Feb 26;6(2):e0005249. doi: 10.1371/journal.pgph.0005249 (PMC12944800; doi:10.1371/journal.pgph.0005249)
Supplement: S1 Appendix — (DOCX) [file pgph.0005249.s001.docx]

**S1 Appendix:**

*Focus Group Guide*

**GENERAL HEALTH**

1. What are your biggest health concerns?
2. Do you feel you have adequate access to healthcare? **Yes or no? If no, please explain.**
3. Have you had any of the following concerns this year?
   1. Fever
   2. Joint pain
   3. Abdominal pain
   4. Diarrhea
   5. Vomiting
   6. Headaches
   7. Rashes
   8. Chest pain
   9. Cough
   10. Blurry vision
   11. Shortness of breath
   12. Malnutrition
   13. Sleep problems
   14. Pregnancy
   15. Dengue
   16. Malaria
   17. Snake bites
   18. Work Accidents (broken bones, cuts, etc.)
   19. Domestic violence
   20. Sexual assault

**HEALTH MAINTENANCE**

1. How many times have you visited your community health worker **within the last year?**
2. **Not at all**
3. **1-2 times**
4. **3-4 times**
5. **More than 4 times**
6. Do you see your community health worker for regular visits or only if there is a health concern?
7. When is the last time you had your blood pressure checked?
8. **Never**
9. **Within the past year**
10. **More than 1 year ago**
11. Have you had bloodwork done?
12. Have you received any vaccinations? **If yes, do you know what they were for?**
13. Have you ever had a pap smear? Mammogram? Colonoscopy?
14. Have you had an eye exam?
15. If needed, do you know where to get glasses?
16. Have you had your hearing tested?
17. If needed, do you know where to get hearing aids?
18. Where do you get your medications? Do you look at the expiration dates?

**MODERN MEDICINE VS TRADICIONAL MEDICINE**

1. What are your beliefs about western medicine?
2. What are common remedies you use for diarrhea? Headaches? Nausea and vomiting? Etc.
3. Do you rely on any traditional medicines to cure or maintain your health? **If yes, which traditional medicines do you use?**

**NUTRITION**

1. Do you feel as if you have access to enough food on a daily basis?
2. **Yes, all of the time**
3. **Most of the time**
4. **Sometimes**
5. **No**
6. What do you eat in a typical day and how much?
7. Where does your food come from?
8. How do you get your food?
9. **Grow own crops**
10. **Fishing**
11. **Hunting**
12. **Buy in a store**
13. **Other**
14. What types of food are available to you? **For example, fruits, vegetables, fish, chicken, eggs, etc.**
15. Do you know how many calories you get per day?
16. Do you eat packaged or canned foods? Do you drink sodas?
17. Does your diet change during the dry vs rainy season? **If yes, how does it change?**
18. Where does your drinking water come from?
19. Is your drinking water clean or are there problems?
20. Do children have access to rehydration packets when they get diarrhea?
21. **Do you know how to make rehydrating solution when dehydrated? If yes, how do you make it?**

**MATERNAL/INFANT HEALTH**

1. Number of times pregnant? How many children?
2. What kind of care do you get during pregnancy?
3. **No care**
4. **Care at home**
5. **Travel to clinic**
6. **Other-please explain**
7. Were there any complications with the pregnancy or delivery? **For example, high blood pressure, seizures, bleeding, etc.**
8. Who attends births and where do you give birth? **Do you give birth at home, in a hospital/clinic, somewhere else?**
9. What kind of care do you get during and after birth?
10. Did you have any difficulties with breastfeeding?
11. Do you use infant formula instead of breastfeeding?
12. What foods are introduced when weaning off breastfeeding?
13. Any feelings of sadness after giving birth?

**ENVIRONMENTAL FACTORS**

1. Is there enough shelter from rain and heat?
2. Does your land provide enough resources for food and shelter?
3. How do you protect yourself from mosquitos?
4. Do you worry that your food or water is contaminated?
5. What kinds of chemicals are you exposed to in your work or home?

**MALARIA PRACTICES**

1. How familiar are you with the signs and symptoms of malaria in children?
   1. Can you identify common symptoms?
2. Do you know when and where children are at the highest risk of contracting malaria?
3. How do you protect yourself and children from mosquito bites?
4. How important is protecting yourself from mosquitoes?
5. How often do you take these precautions?
6. How serious do you perceive the threat of childhood malaria in your community?
7. Is there anything being done at the community level to reduce mosquitoes or prevent bites?
8. How satisfied are you with these efforts?
9. When a child appears sick with malaria, what do you do?
10. Do you feel like you have access to sufficient resources to help a child sick with malaria?
11. How easy is it for you to access medical facilities or care?
12. Are you satisfied with this access?
13. What barriers do you face in regards to accessing medical care?
14. Any additional thoughts, suggestions, or recommendations?
