# Supplementary material for: Community health knowledge and access to care in post-conflict Northern Uganda: Perspectives of community health workers in Pader District
Source: PLOS Glob Public Health. 2026 Feb 26;6(2):e0005249. doi: 10.1371/journal.pgph.0005249 (PMC12944800; doi:10.1371/journal.pgph.0005249)
Supplement: S2 Appendix — (DOCX) [file pgph.0005249.s002.docx]

**S2 Appendix:**

*Adapted Focus Group Guide*

**Questions rephrased in Acholi to be directed at CHWs asking about their community members**

**GENERAL HEALTH**

1. What are your community’s biggest health concerns? List.
2. Do you feel that people in your community have adequate access to healthcare? **Yes or no? If no, please explain.**

**HEALTH MAINTENANCE**

1. How often do community members come to see you for care?
2. Do you community members come to you (CHWs) for regular visits or only if there is a health concern?
3. How often do community members go for routine bloodwork?
4. Do community members get all of their regular vaccinations?
5. Do community members go in for testing like pap smears, Mammograms, or Colonoscopy?
6. Do community members get routine testing for hearing and vision?
7. Do they know where to go for testing? For new glasses or hearing aids?
8. Where do community members get their medications? Do they look at the expiration dates?

**MODERN MEDICINE VS TRADICIONAL MEDICINE**

1. What are community members’ beliefs about western medicine?
2. What are common remedies they use for diarrhea? Headaches? Nausea and vomiting? Etc.
3. Do community members rely on any traditional medicines to cure or maintain their health? **If yes, which traditional medicines do you use?**

**NUTRITION**

1. Do community members feel as if they have access to enough food on a daily basis?
2. What do people eat in a typical day and how much?
3. Where does food come from?
4. How do community members get their food?
5. What types of food are available to the community? **For example, fruits, vegetables, fish, chicken, eggs, etc.**
6. Do community members eat packaged or processed foods? Like soda?
7. Do peoples’ diets change during the dry vs rainy season? **If yes, how does it change?**
8. Where does drinking water come from?
9. Is the drinking water clean or are there problems?
10. Do children have access to rehydration packets when they get diarrhea?

**MATERNAL/INFANT HEALTH**

1. Number of times pregnant? How many children?
2. What kind of care do women in the community get during pregnancy?
3. How commonly are there any complications with the pregnancy or delivery? **For example, high blood pressure, seizures, bleeding, etc.**
4. Who attends births and where do community members give birth?
5. What kind of care do mothers get during and after birth?
6. How common are difficulties with breastfeeding?
7. Do people use infant formula instead of breastfeeding?
8. What foods are introduced when weaning off breastfeeding?
9. Any feelings of sadness among community members after giving birth?

**ENVIRONMENTAL FACTORS**

1. Is there enough shelter from rain and heat?
2. Does the land provide enough resources for food and shelter?
3. Do people worry that their food or water is contaminated?
4. What kinds of chemicals are people exposed to in work or home?

**MALARIA PRACTICES**

1. How familiar are community members with the signs and symptoms of malaria in children?
   1. Can they identify common symptoms?
2. Do community members know when and where children are at the highest risk of contracting malaria?
3. How do people protect themselves and children from mosquito bites?
4. How important is protecting from mosquitoes?
5. How often do people take these precautions?
6. How serious do community members perceive the threat of childhood malaria in your community?
7. Is there anything being done at the community level to reduce mosquitoes or prevent bites?
8. How satisfied are community members with these efforts?
9. When a child appears sick with malaria, what do people do?
10. Do community members feel like they have access to sufficient resources to help a child sick with malaria?
11. How easy is it for people to access medical facilities or care?
12. Are they satisfied with this access?
13. What barriers do community members face in regards to accessing medical care?

**Any additional thoughts, suggestions, or recommendations for the Program?**
