# Supplementary material for: Community health knowledge and access to care in post-conflict Northern Uganda: Perspectives of community health workers in Pader District
Source: PLOS Glob Public Health. 2026 Feb 26;6(2):e0005249. doi: 10.1371/journal.pgph.0005249 (PMC12944800; doi:10.1371/journal.pgph.0005249)
Supplement: Attachment — Submitted filename: PLOS GPH Response to Reviewers .docx [file pgph.0005249.s005.docx]

Yale School of Medicine

*Section of Critical Care Medicine*

*Department of Pediatrics*

*Department of Pediatrics*

10/22/2025

PLOS Global Public Health

PGPH-D-25-02643

Re: Manuscript Title: “Exploring Local Health Knowledge and Access: Focus Group Findings from Community Health Workers in Pader District, Uganda”

Dear Reviewers,

Thank you for your detailed review of our manuscript [PGPH-D-25-02643], entitled "“Exploring Local Health Knowledge and Access: Focus Group Findings from Community Health Workers in Pader District, Uganda." We appreciate the opportunity to revise this work and resubmit it for consideration for publication in PLOS Global Public Health. We have addressed the reviewers’ comments and made changes to the manuscript (detailed below). We believe that the paper has been strengthened as a result, and hope that you agree. Please let us know if you have further questions or comments.

Sincerely,

Daniel S. Ebbs DO, MS, FAAP

Section of Pediatric Critical Care Medicine

Department of Pediatrics

Yale University and Yale New Haven Hospital

New Haven, Connecticut

[Daniel.Ebbs@yale.edu](mailto:Daniel.Ebbs@yale.edu)

| Section | Line Reference (approx.) | Reviewer Comment | Changes Made |
| --- | --- | --- | --- |
| Title & Abstract | Lines 1–22 | Clear and descriptive, but the abstract emphasizes findings over methodology. IPA is not fully reflected.  *Add a sentence clarifying interpretive focus and analytic depth.* | Lines 2-35  We agree with the reviewer’s comments to reframe the study as qualitative descriptive rather than IPA, and that is reflected throughout the revised manuscript. IPA was removed from Keywords and the Abstract, and replaced with “qualitative descriptive study guided by interpretive principles.” Language was simplified and sentences were restructured in the Abstract to match reviewer’s request for analytic transparency. |
| Keywords | Lines 23–26 | Inclusion of 'IPA' may misrepresent the design.  *Rephrase as 'qualitative descriptive informed by interpretive principles'.*  *There is nothing implementation science-related in this article, so I propose you drop it as a keyword.* | Line 38  IPA was removed from the Keywords. |
| Introduction | Lines 27–55 | The introduction offers a strong contextual background on Northern Uganda’s post-conflict health challenges and the role of CHWs. However, it reads as a problem-oriented background rather than a conceptually grounded or phenomenological framing.  The section does not clearly articulate the *phenomenon under exploration;* that is, what aspect of CHWs’ lived experience or sense-making the study seeks to understand.  While the authors state in the methods section that the study used an *Interpretive Phenomenological Analysis (IPA)* approach, the framing and objectives (e.g., identifying local health priorities, barriers, and recommendations) are more descriptive and evaluative in nature. This positioning aligns more closely with a **descriptive qualitative design** than with an interpretive phenomenological study, which typically focuses on participants’ *meaning-making processes*.  ***Revise the introduction to clarify the study’s focus and methodological positioning***.  1. If the authors intend to maintain IPA, the introduction should explicitly define the *phenomenon of interest* (e.g., how CHWs experience and make sense of their roles and constraints) and connect it to interpretive or phenomenological theory (e.g., lived experience, identity, agency, or moral responsibility).  2. In either case, include 2–3 sentences articulating the study's conceptual grounding, for instance: *“This study explores how community health workers perceive and describe the health needs and challenges in their communities, using a descriptive qualitative design guided by interpretive principles.”* | Lines 43-47, 56-63, 68-88  More contextual information was added to reflect the positioning of the paper as a qualitative descriptive study. References 3 and 4 were swapped due to the added information. Some wording and sentence structure was changed for clarity and corrected grammar. |
| Study Design & Setting | Lines 56–70 | The authors mention using IPA but employ focus group discussions (FGDs), which are unsuitable for individual-level analysis. The design and data collection are more consistent with a descriptive qualitative approach. ***Reframe as a qualitative descriptive study informed by interpretive principles or justify the use of FGDs within an adapted IPA framework (e.g., collective sense-making).*** Also, the authors explored numerous thematic areas in the FGDs, including general health, health maintenance, nutrition, maternal and infant health, environmental factors, traditional medicine, and malaria practices. While each is individually relevant, the overall scope is overly broad and diffuses the analytic focus, making it unclear what central phenomenon the study sought to explore. The rationale for selecting these domains is not explained, nor is there an indication of a guiding theoretical or conceptual framework. ***Given the wide range of domains explored, the authors could enhance analytic coherence by distilling the data into a few cross-cutting interpretive themes that reflect CHWs' shared experiences. Applying an organizing framework (e.g., the socioecological model) could help integrate these findings around common meanings.*** | Line 91, 103-110  In the Methods section, we reframed the study as qualitative descriptive informed by interpretive principles. |
| Study Population | Lines 71–77 | The manuscript notes that CHWs were **purposively selected** from communities where the **Laro Kwo Project** operates, which is appropriate for qualitative inquiry. However, the rationale for conducting **six FGDs** is not provided, and it remains unclear how the authors determined that the data collected were **sufficient** to capture the range of experiences (i.e., data saturation). Authors should clarify **why six FGDs were considered adequate**. For example, explain whether data collection stopped when no new ideas emerged or whether the number of groups was predetermined based on feasibility or precedent. Add a sentence such as: *Six FGDs were conducted purposively across project communities; data collection continued until no new codes or insights emerged, indicating thematic saturation.* | Lines 115-120  Justification for the number of FGDs was added. |
| Data Collection | Lines 78–95 | The description of data collection is clear and indicates that FGDs were conducted by trained facilitators with local translation, which strengthens contextual validity. However, the section lacks sufficient **reflexivity** on the influence of researcher identities and facilitation dynamics.  Key procedural details are also missing: (1) the **duration** of each FGD is not stated; (2) it is unclear how **group moderation** was managed to ensure all participants contributed equally; and (3) while detailed notes were reportedly taken, **note-taking alone** can be insufficient for capturing verbatim data, especially in multi-participant discussions. It is not indicated whether **audio recordings** were made or how data accuracy and completeness were ensured.  Add a **reflexivity paragraph** outlining how the research team’s positionality (local vs. external researchers) and facilitation approach may have influenced participation or interpretation.  Provide missing procedural details: specify the **average duration of each FGD**, describe **how moderators encouraged participation from quieter members**, and clarify whether **sessions were audio recorded and transcribed verbatim**. If only detailed notes were used, explain how note-takers ensured completeness and accuracy (e.g., simultaneous translation checks, debriefing immediately after sessions). | Lines 129-139  Reflexivity on the influence of researcher identities and details regarding facilitation dynamics were added. Clarification regarding how the FGD guide was adapted and implemented was added. |
| FGD Guide | Lines 86–89; Appendix 1 | The attached FGD guide appears **incongruent with the stated study participants (CHWs)**. Several sections (e.g., *Health Maintenance*, *General Health*, *Nutrition*) contain questions clearly directed at **community members or patients**, such as frequency of visiting a community health worker, personal vaccination history, or access to medical checkups. These questions do not align with the study’s stated aim to explore **CHWs’ perceptions, experiences, and challenges** in their work.  Additionally, the guide is **overly structured and includes many closed or checklist-style questions**, which restrict opportunities for open reflection and depth of response. The format more closely resembles a **community health survey or needs assessment** rather than a qualitative instrument designed for interpretive exploration.  *The authors should* ***clarify which version of the FGD guide was actually used with CHWs****. If the attached tool was adapted from a community survey, specify the adaptation process (e.g., removing or rephrasing patient-oriented questions).*  *If this tool was indeed used with CHWs, acknowledge this as a* ***methodological limitation****, as it likely constrained participants’ ability to reflect on their experiences as health workers. Describe any* ***strategies used to elicit CHWs’ interpretive input,*** *for example, through follow-up probes, reframing of questions during facilitation, or additional open-ended prompts.*  *Finally, consider including a* ***summary of the final FGD guide*** *used in the study (highlighting key domains and sample open-ended questions) to ensure transparency and alignment with the study objectives.* | Lines 144-156, appendix 2  Clarification regarding how the FGD guide was adapted and implemented was added. A sample of the final adapted guide is included with the revised submission. |
| Data Analysis | Lines 96–106 | Coding process linear and descriptive; lacks interpretive iteration.  Clarify analytic approach and include codebook summary or analytic memo. | Lines 158-179  We have clarified the analytic approach and added mention of consent and handling of sensitive topics. |
| Ethical Considerations | Lines 107–114 | Consent procedures are clear but lack mention of handling sensitive topics.  Add a statement on participant support or referral for distress. | Lines 184-188  Statements regarding consent procedures were added. |
| Results Overview | Lines 115–131 | Themes mirror guide domains; limited inductive emergence.  Reframe results around overarching interpretive themes. | Lines 190-192, 200-214, Table 2  Program Feedback has been removed entirely in line with suggestions. Results were reframed around more interpretive themes, and accompanying details were added. |
| Community Health Concerns | Lines 132–150 | Rich narrative but limited interpretive engagement.  Add reflection on emotional/identity aspects of CHWs’ experiences. | Lines 217-228, 235-244  Additional context and reflection were included. |
| Healthcare Access | Lines 151–172 | Strong empirics, but some phrasing (e.g., 'too impoverished') needs tone adjustment.  Use neutral phrasing ('lack financial means') unless quoted directly. | Line 245-263, 271, 287-291  Tone was adjusted for neutral framing. |
| Knowledge vs Practice | Lines 183–223 | Descriptive section: outdated term 'villagers'.  Replace with 'community members' and deepen interpretive reflection. | Line 293, 317, 333-335  Deeper interpretation added and terms changed |
| Feedback for Project | Lines 224–275 | This section presents detailed operational feedback from participants directed at the *Laro Kwo Project,* for example, requests for supplies, training, supervision, and incentives. While these reflections are valuable for internal program improvement, they are not directly relevant to an academic manuscript intended for global readership. Their inclusion shifts the tone of the paper toward a project report rather than a scholarly analysis.  Authors should consider removing this **as a standalone component** and instead integrating key insights **into the Discussion section**. | Lines 337-389  Removed this section entirely. |
| Discussion | Lines 277–333 | The discussion summarizes well the study’s main findings and situates them within the broader literature on community health in low-resource settings. However, it largely reiterates well-known challenges, such as limited supplies, stockouts, poor infrastructure, and inadequate supervision, without offering new analytic or contextual insights. As a result, the section reads more as an extension of the results than as an interpretive discussion.  The discussion could be strengthened by focusing on **how these familiar barriers manifest in the specific context of Pader District** and what they reveal about the functioning and lived realities of CHWs in post-conflict rural Uganda. Rather than repeating findings, the authors could elaborate on **why** these challenges persist, **how** CHWs navigate them, and **what implications** arise for sustaining CHW motivation and community trust. | Lines 391-556  The Discussion was rewritten from reiterating results to providing interpretive analysis of the situation specific to the CHWs in Pader. |
