# Supplementary material for: Community health knowledge and access to care in post-conflict Northern Uganda: Perspectives of community health workers in Pader District
Source: PLOS Glob Public Health. 2026 Feb 26;6(2):e0005249. doi: 10.1371/journal.pgph.0005249 (PMC12944800; doi:10.1371/journal.pgph.0005249)
Supplement: Attachment — Submitted filename: Response to Reviewers PLOS GPH 02022026.docx [file pgph.0005249.s007.docx]

02/02/2026

PLOS Global Public Health

Response to Reviewers for PGPH-D-25-02643R1

Manuscript Title: “Exploring Local Health Knowledge and Access: Focus Group Findings from Community Health Workers in Pader District, Uganda”

Dear Reviewers,

Thank you for your time reviewing my submission and I appreciate all of your comments and suggestions.

First, I agree with the reviewer’s comment to revise the title for clarity on what and whose knowledge was explored in the study. The revised title is “Community Heath Knowledge and Access to Care in Post-Conflict Northern Uganda: Perspectives of Community Health Workers in Pader District”. The rest of the edits in response to reviewer comment are listed below:

| **Section** | **Line Ref.** | **Reviewer Comment** | **Changes Made** |
| --- | --- | --- | --- |
| **Title** | Title | 1. Please clarify which knowledge was explored in this study? For example, knowledge of the common health problems, causes, prevention, access to care, etc.?  2. How was knowledge explored? Were you finding out how CHWs understand the common health problems, their causes, prevention, and management? Or did the study explore what CHWs know about the community’s knowledge and access to health services? In other words, was the study exploring knowledge among CHWs or knowledge among communities where CHWs come from? | - All aspects of health knowledge were explored – the most common issues, access to care, treatment and prevention behaviors, and social determinants of health - Full title revised to “Community Heath Knowledge and Access to Care in Post-Conflict Northern Uganda: Perspectives of Community Health Workers in Pader District” |
| **Abstract** | 3-4 | Clarify if community health workers in this study include village health teams (VHTs), community health extension workers (CHEWs), or both | Line 5-9 added; clarified involvement of CHWs and VHTs |
|  | 9 | If the title is focusing on local health knowledge and access, should the methodology be focused on exploring perspectives and experiences? How about if the study had focused on exploring the CHW’s understanding of the local health problems and access to healthcare services for the identified health problems? | Line 21-22; clarification added throughout abstract that study explored CHWs’ understanding of community member experience and perspectives on health and access to care |
|  | 10 | Indicate the period during which the group discussions were conducted. | Line 16; study period added |
|  | 11 | How did you arrive at the eight domains? Are they adapted from another source? If so, then specify the source. If the researchers developed the domains, please indicate the criteria used to create them. Given that the FGD was developed based on the domains, should we take it that the inquiry was based on a priori themes? | Lines 19-21; clarification of how FGD guide was developed |
|  | 15 | How are the three themes related to the eight domains? | Line 27; connected themes to domains |
|  | 21 | What does strong awareness mean? | Clarified, Line 32-33 |
|  | 22-28 | Some findings may be left out since they are not related to the title of this manuscript… | Lines 35-43; Rephrase and clarification added |
| **Intro** | 37-40 | Provide Citation | References added |
|  | 55 | Define what CHWs are. Show reference to WHO and the National policies. In the case of Uganda, specify if you are including VHTs and CHEWs among CHWs. | Lines 82-92; definitions added for CHWs and VHTs |
|  | 67 | Harmonize knowledge gap and objectives | Lines 101-114; harmonized the knowledge gap and objectives |
| **Methods** | 79-83 | Additional details on CHW program and training | Lines 122-127; details added |
|  | 85 | “…The in-depth understanding of CHWs' roles and experiences within their communities and the broader health system context…” does not match well with the focus of the study. It would be better to describe the methodology in relation to the title. For example, “….in-depth understanding of CHWs' knowledge of the community health problems…” | Line 147 |
|  | 88 | It remains unclear how the eight domains were used to explore the local health knowledge and access. | Lines 153-156; clarified |
|  | 110 | Clarification for final adapted focus group guide | Appendix 2 |
|  | 118-120 | What does “alongside” mean? Did the program coordinator participate in the FGDs? | Line 192; clarified |
| **Results** | 161-163 | Harmonize language of objectives with results | Lines 236-244; harmonized throughout intro, methods, results |
|  | 172-174 | Harmonize language of themes within results and abstract | Lines 27-29; harmonized across intro, methods, results |
|  | Table 2 | See comments | Table 2; some wording changed and added |
|  | Quotations | Formatting | Fixed throughout Results section |
|  | 198-199 | “Others linked these struggles to the lasting trauma of the conflict period, suggesting that grief, displacement, and hardship have left lingering effects on community wellbeing.” It is not clear how the difficulty in accessing health services is being linked to the lasting trauma of the conflict period. Was this finding well validated with strategies like audit trail, member checks and triangulation? Secondly, avoid sentiments like “these struggles” since this is the researcher’s language. Not a quotation of the participant. | Lines 310-311; fixed |
|  | 202-204 | “CHWs described long distances to facilities, typically 5–7 kilometers, sometimes up to 20, and the absence of affordable transport options. Hospitals were even farther, often unreachable during emergencies.” Distinguish between the hospitals and facilities in the sentences | Lines 315-318 |
|  | 276-277 | “As trusted figures within their communities, CHWs increasingly serve as informal first points of contact for basic care and health education.” This sounds like an interpretation or discussion of results, which should not be in this section. | Line 443-446; removed |
| **Discussion** | 285-287 | “The findings illustrate how CHWs operate within a health landscape shaped by structural fragility, poverty, and the enduring effects of conflict, yet also demonstrate their adaptability, resilience, and strong sense…” Are you sure the findings of this study demonstrate these? | Lines 467-476; wording changed |
|  | 289 | Started with Barriers paragraph instead of health knowledge | Paragraphs swapped |
|  | 291 | “…their manifestation in Pader District reflects a distinct post-conflict reality.” Clarify what you mean here. Do you mean the manifestation of these challenges in Pader district are due to the post-conflict situation? These issues seem to be the general challenges facing the health sector in the rest of Uganda irrespective of the post-conflict situation. | Lines 523-531; clarification added |
|  | 301-302 | “The consistent availability of diagnostic and treatment tools remains elusive…” Use a simple and neutral tone. Avoid words like elusive unless they really add value to what you aim to communicate. | Line 480; tone adjusted |
|  | 304-305 | Use simple and neutral tone | Lines 477-486; tone fixed |
|  | 326-328 | “The mismatch between health knowledge and practice among community members further underscores the role of CHWs as navigators of both biomedical and socioeconomic realities.” Not clear. Do you mean the mismatch shows the role of CHWs? So, how does the role of CHWs solve the issues that are responsible for the mismatch? | Lines 558-568; clarified |
|  | 330 | Provide citation | Line 565 |
|  | 331-332 | “The CHWs’ insights highlight that education alone cannot overcome material deprivation and that health behavior is inseparable from economic circumstance.” This study alone cannot make you arrive at this conclusion with certainty. | Lines 558-568; wording adjusted |
|  | 335 | “The dual burden carried by CHWs, being both trusted and under-resourced providers.” Is this accurate? Being under-resourced may be a burden, but should being trusted also be a burden? Please clarify. | Lines 569-592; clarified |
|  | 337 | “…often absorbing community frustration…” Provide a citation for this statement. Should this study alone conclude that “CHWs are often absorbing community frustration”? | Lines 569-592 |
|  | 348-350 | “…attention to the social and relational dimensions of care . First, ensuring reliable supply chains for diagnostics and essential medicines remains fundamental, particularly in geographically isolated areas such as Pader District…” Which results of this study relates to social and relational dimensions of care? How do these relate with social and relational dimensions of care? How does “reliable supply chains for diagnostics and essential medicines” relate with social and relational dimensions of care? | Changes made to discussion section |
|  | 352-356 | Tie discussion to results | Lines 595-611 |
|  | 358-361 | Tie discussion to results | Lines 595-611 |
| **Conclusion** | 378-379 | “Community health workers in Pader District play a vital role in addressing persistent health challenges in rural Northern Uganda…” This was not a finding nor a focus of the study. Therefore, consider removing this statement from the conclusion. | Lines 643-687 |
|  | 383-392 | The recommendations should be focused on local health knowledge and access. CHWs can be part of that. | Lines 643-687 |
