# Supplementary material for: Community health knowledge and access to care in post-conflict Northern Uganda: Perspectives of community health workers in Pader District
Source: PLOS Glob Public Health. 2026 Feb 26;6(2):e0005249. doi: 10.1371/journal.pgph.0005249 (PMC12944800; doi:10.1371/journal.pgph.0005249)
Supplement: Attachment — Submitted filename: Reviewer Comment.docx [file pgph.0005249.s004.docx]

**Peer Review Report**

Manuscript Title: Exploring Local Health Knowledge and Access: Focus Group Findings from Community Health Workers in Pader District, Uganda

Journal: PLOS Global Public Health

Reviewer Summary and Recommendation

This manuscript addresses a critical topic in community health and provides valuable insights into the lived realities of community health workers (CHWs) in post-conflict Northern Uganda. The study is well-contextualized, ethically grounded, and relevant for health systems strengthening. However, while it claims to employ Interpretive Phenomenological Analysis (IPA), the methodology and results align more closely with a qualitative descriptive design. The focus group format, structured guide, and domain-driven analysis limit interpretive depth. Greater attention to reflexivity, analytic transparency, and phenomenological interpretation is required to align with the stated methodological orientation.

Overall Recommendation: Major Revision

The paper should be reframed as a qualitative descriptive study informed by interpretive principles. The authors should expand interpretive depth, include reflexivity, provide analytic traceability (e.g., codebook summary), and adjust tone for inclusivity and neutrality.

**Summary of Major and Minor Revisions**

| Revision Category | Focus Areas |
| --- | --- |
| Major Revisions | Reframe the study as qualitative descriptive rather than IPA.  Expand interpretive depth in results and discussion.  Add reflexivity and address positionality.  Where possible, provide a codebook as part of the attachments for analytical transparency.  Reorganize results around interpretive themes.  Clarify methodological justification for FGDs. |
| Minor Revisions | Adjust language for inclusivity (replace 'villagers', 'too impoverished').  Enhance variety in reporting verbs.  Add a discussion of the ethical handling of sensitive topics.  Provide more concise and varied abstract phrasing.  Strengthen the link between findings and theory. |
| Minor Comment | Please verify and update the professional qualifications of some co-authors. For instance, other publications list Felix *Bongomin* as holding additional qualifications (e.g., **MMED**). |

**Detailed Reviewer Critique by Manuscript Section**

| Section | Line Reference (approx.) | Reviewer Comment |
| --- | --- | --- |
| Title & Abstract | Lines 1–22 | Clear and descriptive, but the abstract emphasizes findings over methodology. IPA is not fully reflected.  *Add a sentence clarifying interpretive focus and analytic depth.* |
| Keywords | Lines 23–26 | Inclusion of 'IPA' may misrepresent the design.  *Rephrase as 'qualitative descriptive informed by interpretive principles'.*  *There is nothing implementation science-related in this article, so I propose you drop it as a keyword.* |
| Introduction | Lines 27–55 | The introduction offers a strong contextual background on Northern Uganda’s post-conflict health challenges and the role of CHWs. However, it reads as a problem-oriented background rather than a conceptually grounded or phenomenological framing.  The section does not clearly articulate the *phenomenon under exploration;* that is, what aspect of CHWs’ lived experience or sense-making the study seeks to understand.  While the authors state in the methods section that the study used an *Interpretive Phenomenological Analysis (IPA)* approach, the framing and objectives (e.g., identifying local health priorities, barriers, and recommendations) are more descriptive and evaluative in nature. This positioning aligns more closely with a **descriptive qualitative design** than with an interpretive phenomenological study, which typically focuses on participants’ *meaning-making processes*.  ***Revise the introduction to clarify the study’s focus and methodological positioning***.  1. If the authors intend to maintain IPA, the introduction should explicitly define the *phenomenon of interest* (e.g., how CHWs experience and make sense of their roles and constraints) and connect it to interpretive or phenomenological theory (e.g., lived experience, identity, agency, or moral responsibility).  2. In either case, include 2–3 sentences articulating the study's conceptual grounding, for instance: *“This study explores how community health workers perceive and describe the health needs and challenges in their communities, using a descriptive qualitative design guided by interpretive principles.”* |
| Study Design & Setting | Lines 56–70 | The authors mention using IPA but employ focus group discussions (FGDs), which are unsuitable for individual-level analysis. The design and data collection are more consistent with a descriptive qualitative approach. ***Reframe as a qualitative descriptive study informed by interpretive principles or justify the use of FGDs within an adapted IPA framework (e.g., collective sense-making).*** Also, the authors explored numerous thematic areas in the FGDs, including general health, health maintenance, nutrition, maternal and infant health, environmental factors, traditional medicine, and malaria practices. While each is individually relevant, the overall scope is overly broad and diffuses the analytic focus, making it unclear what central phenomenon the study sought to explore. The rationale for selecting these domains is not explained, nor is there an indication of a guiding theoretical or conceptual framework. ***Given the wide range of domains explored, the authors could enhance analytic coherence by distilling the data into a few cross-cutting interpretive themes that reflect CHWs' shared experiences. Applying an organizing framework (e.g., the socioecological model) could help integrate these findings around common meanings.*** |
| Study Population | Lines 71–77 | The manuscript notes that CHWs were **purposively selected** from communities where the **Laro Kwo Project** operates, which is appropriate for qualitative inquiry. However, the rationale for conducting **six FGDs** is not provided, and it remains unclear how the authors determined that the data collected were **sufficient** to capture the range of experiences (i.e., data saturation). Authors should clarify **why six FGDs were considered adequate**. For example, explain whether data collection stopped when no new ideas emerged or whether the number of groups was predetermined based on feasibility or precedent. Add a sentence such as: *Six FGDs were conducted purposively across project communities; data collection continued until no new codes or insights emerged, indicating thematic saturation.* |
| Data Collection | Lines 78–95 | The description of data collection is clear and indicates that FGDs were conducted by trained facilitators with local translation, which strengthens contextual validity. However, the section lacks sufficient **reflexivity** on the influence of researcher identities and facilitation dynamics.  Key procedural details are also missing: (1) the **duration** of each FGD is not stated; (2) it is unclear how **group moderation** was managed to ensure all participants contributed equally; and (3) while detailed notes were reportedly taken, **note-taking alone** can be insufficient for capturing verbatim data, especially in multi-participant discussions. It is not indicated whether **audio recordings** were made or how data accuracy and completeness were ensured.  Add a **reflexivity paragraph** outlining how the research team’s positionality (local vs. external researchers) and facilitation approach may have influenced participation or interpretation.  Provide missing procedural details: specify the **average duration of each FGD**, describe **how moderators encouraged participation from quieter members**, and clarify whether **sessions were audio recorded and transcribed verbatim**. If only detailed notes were used, explain how note-takers ensured completeness and accuracy (e.g., simultaneous translation checks, debriefing immediately after sessions). |
| FGD Guide | Lines 86–89; Appendix 1 | The attached FGD guide appears **incongruent with the stated study participants (CHWs)**. Several sections (e.g., *Health Maintenance*, *General Health*, *Nutrition*) contain questions clearly directed at **community members or patients**, such as frequency of visiting a community health worker, personal vaccination history, or access to medical checkups. These questions do not align with the study’s stated aim to explore **CHWs’ perceptions, experiences, and challenges** in their work.  Additionally, the guide is **overly structured and includes many closed or checklist-style questions**, which restrict opportunities for open reflection and depth of response. The format more closely resembles a **community health survey or needs assessment** rather than a qualitative instrument designed for interpretive exploration.  *The authors should* ***clarify which version of the FGD guide was actually used with CHWs****. If the attached tool was adapted from a community survey, specify the adaptation process (e.g., removing or rephrasing patient-oriented questions).*  *If this tool was indeed used with CHWs, acknowledge this as a* ***methodological limitation****, as it likely constrained participants’ ability to reflect on their experiences as health workers. Describe any* ***strategies used to elicit CHWs’ interpretive input,*** *for example, through follow-up probes, reframing of questions during facilitation, or additional open-ended prompts.*  *Finally, consider including a* ***summary of the final FGD guide*** *used in the study (highlighting key domains and sample open-ended questions) to ensure transparency and alignment with the study objectives.* |
| Data Analysis | Lines 96–106 | Coding process linear and descriptive; lacks interpretive iteration.  Clarify analytic approach and include codebook summary or analytic memo. |
| Ethical Considerations | Lines 107–114 | Consent procedures are clear but lack mention of handling sensitive topics.  Add a statement on participant support or referral for distress. |
| Results Overview | Lines 115–131 | Themes mirror guide domains; limited inductive emergence.  Reframe results around overarching interpretive themes. |
| Community Health Concerns | Lines 132–150 | Rich narrative but limited interpretive engagement.  Add reflection on emotional/identity aspects of CHWs’ experiences. |
| Healthcare Access | Lines 151–172 | Strong empirics, but some phrasing (e.g., 'too impoverished') needs tone adjustment.  Use neutral phrasing ('lack financial means') unless quoted directly. |
| Knowledge vs Practice | Lines 183–223 | Descriptive section: outdated term 'villagers'.  Replace with 'community members' and deepen interpretive reflection. |
| Feedback for Project | Lines 224–275 | This section presents detailed operational feedback from participants directed at the *Laro Kwo Project,* for example, requests for supplies, training, supervision, and incentives. While these reflections are valuable for internal program improvement, they are not directly relevant to an academic manuscript intended for global readership. Their inclusion shifts the tone of the paper toward a project report rather than a scholarly analysis.  Authors should consider removing this **as a standalone component** and instead integrating key insights **into the Discussion section**. |
| Discussion | Lines 277–333 | The discussion summarizes well the study’s main findings and situates them within the broader literature on community health in low-resource settings. However, it largely reiterates well-known challenges, such as limited supplies, stockouts, poor infrastructure, and inadequate supervision, without offering new analytic or contextual insights. As a result, the section reads more as an extension of the results than as an interpretive discussion.  The discussion could be strengthened by focusing on **how these familiar barriers manifest in the specific context of Pader District** and what they reveal about the functioning and lived realities of CHWs in post-conflict rural Uganda. Rather than repeating findings, the authors could elaborate on **why** these challenges persist, **how** CHWs navigate them, and **what implications** arise for sustaining CHW motivation and community trust. |
